# Cationic Nanostructures against Foodborne Pathogens

**DOI:** 10.3389/fmicb.2016.01804

**Published:** 2016-11-09

**Authors:** Letícia Dias de Melo Carrasco, Ronaldo Bertolucci, Rodrigo T. Ribeiro, Jorge L. M. Sampaio, Ana M. Carmona-Ribeiro

**Affiliations:** ^1^Laboratório de Biocolóides, Departamento de Bioquímica, Instituto de Química, Universidade de São PauloSão Paulo, Brazil; ^2^Laboratório de Microbiologia, Departamento de Análises Clínicas e Toxicológicas, Faculdade de Ciências Farmacêuticas, Universidade de São PauloSão Paulo, Brazil

**Keywords:** gramicidin D, dioctadecyldimethylammonium bromide, polydiallyldimethyl ammonium chloride, polymethylmethacrylate, *Escherichia coli*, *Staphylococcus aureus*, *Salmonella enterica*, *Listeria monocytogenes*

In food microbiology, novel strategies to fight foodborne pathogens are certainly welcome. In this data report, cationic nanostructures built from combinations of nanoparticles, antimicrobial peptide and cationic lipid are evaluated against important foodborne pathogens such as *Escherichia coli, Salmonella enterica* subsp. serovar Typhymurium, *Staphylococcus aureus* and *Listeria monocytogenes.*

The cationic lipid dioctadecyldimethylammonium bromide (DODAB), the antimicrobial peptide gramicidin D (Gr), the antimicrobial cationic polymer poly (diallyldimethylammonium chloride) (PDDA) and the biocompatible polymer poly (methyl methacrylate) (PMMA) can be combined to yield a variety of antimicrobial cationic nanostructures as previously described by our group (Carmona Ribeiro and Chaimovich, 1983; Martins et al., 1997; Lincopan et al., 2003, 2005; Pereira et al., 2008; Melo et al., 2010, 2011; Carvalho et al., 2012; Naves et al., 2013; Ragioto et al., 2014; Carrasco et al., 2015; Sanches et al., 2015). However, these nanostructures were not specifically evaluated against foodborne pathogens before. This data report aims at filling up this gap.

The cationic lipid (DODAB) and the cationic polymer PDDA bear quaternary antimicrobial nitrogens and form a variety of cationic nanostructures as the closed or open bilayers; the hybrid polymeric nanoparticles NPs and the DODAB/Gr combinations. Schemes, physical properties and antimicrobial activity for the cationic assemblies against the foodborne pathogens are on the data set (https://www.researchgate.net/publication/308140571_September_15_2016_data_set_on_cationic_assemblies_against_food_pathogens.)

DODAB, Gr, PDDA, PMMA, ethanol, 2,2,2-trifluoroethanol (TFE) and NaCl were from Sigma-Aldrich (St. Louis, MO, USA). DODAB LV were obtained by hydrating and vortexing the DODAB powder in 1 mM NaCl aqueous solution, at 60°C at 2 mM DODAB (Carmona Ribeiro and Chaimovich, 1983). For obtaining the DODAB BF, LV were ultrasonically disrupted with a macrotip (85 W/15 min/70°C) before centrifuging (10,000 rpm/60 min/4°C) and collecting the supernatant (Carmona-Ribeiro, 2006). DODAB analysis was via microtitration of its bromide counterion (Schales and Schales, 1941).

A Gr stock solution (6.4 mM Gr) in TFE was added to previously prepared LV or BF at a 1:10 Gr:DODAB molar ratio. DODAB/Gr dispersions were prepared from DODAB LV incubated for 1 h/60°C with Gr (Ragioto et al., 2014). DODAB LV/ Gr sonicated with macrotip (85 W/15 min/70°C) and centrifuged (10,000 rpm/60 min/4°C) yield the DODAB BF/Gr.

PMMA/DODAB and PMMA/PDDA NPs were obtained as previously described (Naves et al., 2013; Sanches et al., 2015).

All nanostructures were characterized for sizing (zeta-average diameter or Dz), zeta-potential (ζ) and polydispersity (P) using a Zeta Plus-Zeta Potential Analyzer (Brookhaven Instruments Corporation, Holtsville, NY, USA) equipped with a laser (677 nm) for DLS with measurements at 90° (Grabowski and Morrison, 1983). ζ values were calculated from the electrophoretic mobility (μ) and Smoluchovski equation (ζ = μη / ε, where η is the medium viscosity and ε, the dielectric constant).

Food pathogens from American Type Culture Collection (ATCC) (Manassas, VA, USA) were *S. aureus* ATCC 29213, *E. coli* ATCC 25922, *L. monocytogenes* ATCC 19111 and *S. enterica* ATCC 14028. After reactivation from frozen stocks in MHA, strains' cultures incubated in MHA (37°C/18–48 h depending on the pathogen) had some colonies transferred to a 1 mM NaCl solution and turbidity was adjusted to 0.5 McFarland (Chapin and Lauderdale, 2007). After 1 h interaction between nanostructures and bacteria in 1 mM NaCl over a range of DODAB, Gr or PDDA concentrations in the nanostructures, mixtures were diluted up to 100,000 before plating 0.1 mL onto MHA surface in triplicate. Controls were bacteria only in 1 mM NaCl (plated after 1 h). After incubation (37°C/24–48 h) and CFU/mL counting, MBC is the lowest concentration yielding the minimal CFU counting.

Gr insertion in DODAB LV bilayer reduced Dz and increased the positive ζ-potential. Gr tryptophans anchoring the peptide at the bilayer-water interface sterically stabilized DODAB/Gr. Disrupting DODAB LV/Gr led to cationic bilayers with Gr molecules inserted as dimeric channels so that the packing of the cationic lipids in the bilayer and the ζ-potentials increased. Other assemblies also tested in this work against the food pathogens had DODAB embedded in PMMA or PDDA making an outer layer (shell) in core-shell PMMA/PDDA positively charged NPs.

DODAB not only carried Gr but also displayed antimicrobial activity and reduced the MBC values against most strains tested. Table 1 shows MBCs in mM or mg/mL and the total reduction in viability caused by the antimicrobials. The Gr peptide was effective against the two Gram-positive bacteria. DODAB BF or LV affected all bacteria tested with exception of *S. enterica.* Mostly DODAB BF was more efficacious against the bacteria than LV. The Gr peptide in DODAB BF reduced MBC values against three bacteria strains (lines 2 and 3, Table 1). This effect was important due to the toxicity of the cationic lipid and the antimicrobial peptide. *S. enterica* was the most refractory strain to the cationic agents alone or in combinations with exception of PDDA or PMMA/PDDA NPs (Table 1). In particular, the PMMA/PDDA NPs (last line on Table 1) were very efficient against *S. enterica.* DODAB in the PMMA/DODAB NPs displayed a reduced antimicrobial activity whereas PDDA exposure as an outer shell on the PMMA/PDDA NPs increased the antimicrobial activity (Table 1). The log of viability reduction at MBC against *S. enterica* of DODAB BF/Gr and DODAB LV/Gr was slightly lower than the one for DODAB BF and DODAB LV possibly due to the bulky nature of Gr tryptophans located at the bilayer/water interface, which prevented the close electrostatic attraction between the cationic moieties of the nanostructures and the anionic moieties of the bacteria.

**Table 1:** **MBC (mM; mg/mL) and log of viability reduction at MBC for cationic nanostructures against food pathogens**.

**Assembly**	**MBC in mM; mg/mL/ Reduction in log(CFU/mL)**			
	***E. coli***	***S. enterica***	***S. aureus***	***L. monocytogenes***
Gr	0.010; 0.019/ 0.3	0.010; 0.019/ 0.5	0.010; 0.019/ 2.1	0.005; 0.009/ 7.6
DODAB BF	0.063; 0.039/ 7.6	0.500; 0.316/ 1.3	0.063; 0.039/ 3.4	0.125; 0.079/ 7.8
DODAB BF/Gr	0.031; 0.019/ 7.5	0.250; 0.158/ 0.9	0.015; 0.010/ 3.8	0.125; 0.079/ 8.0
DODAB LV	0.015; 0.010/ 4.5	0.500; 0.316/ 0.7	0.015; 0.010/ 2.9	0.250; 0.158/ 5.7
DODAB LV/Gr	0.015; 0.010/ 4.6	0.500; 0.316/ 0.4	0.031; 0.019/ 2.7	0.063; 0.039/ 6.0
PMMA/DODAB	------; 2.500/ 2.2	------; 1.250/ 0.1	------; 5.000/ 3.1	------; 5.000/ 1.5
PDDA	------; 0.005/ 7.5	------; 4.810/ 3.3	------; 0.010/ 5.8	------; 0.048/ 0.5
PMMA/PDDA	------; 0.009/ 7.5	------; 0.940/ 6.9	------; 0.940/ 7.1	------; 0.940/ 5.1

For DODAB/Gr combinations, the molar ratio is [Gr] = 0.1[DODAB].

Antimicrobial activity can be determined as inhibition of growth or minimal inhibitory concentration (MIC) or as cells survival in log (CFU/mL) (MBC). Here MBC determinations and reduction in log(CFU/mL) at MBC properly quantified the antimicrobial effect of the cationic nanostructures (Table 1). As concentrations required for inhibition are smaller than those for death, the consistency of the results can be checked: MIC for Gr against *S. aureus* was 2.5 μM. (Wang et al., 2012) and MBC for Gr against *S. aureus* was 10 μM (Table 1), a value consistently higher than the MIC value. Gr displayed a high toxicity against mammalian (Sorochkina et al., 2012; Wang et al., 2012) and eukaryotic cells such as *S. cerevisae* seen as 50% of cell viability at 1 μM Gr (Ragioto et al., 2014). However, in formulations with DODAB, Gr toxicity decreased against *S. cerevisae* (Ragioto et al., 2014). Reductions in MBC for DODAB in the combinations with Gr mean reduction in Gr doses since Gr concentration is always 10% of the DODAB concentration in each combination. Against mammalian cells, 0.5 mM DODAB killed 50% of fibroblasts in culture (Carmona-Ribeiro et al., 1997). Despite the DODAB relative toxicity *in vitro*, there were instances of good activity for DODAB formulations *in vivo*. For example, DODAB could be used as an effective immunoadjuvant in combination with peptides or proteins for vaccines (Tsuruta et al., 1997; Carmona-Ribeiro, 2014) or could incorporate amphotericin B against systemic candidiasis in mice inducing about 100% of mice survival after treatment in absence of nephrotoxicity (Lincopan et al., 2003, 2005).

DODAB and DODAB/Gr interacted with bacteria driven by the electrostatic attraction and their mechanism of action involved lysis of the bacteria with leakage of intracellular compounds to the external medium and distortions in cell morphology (Martins et al., 1997; Ragioto et al., 2014). Gr required insertion in the bacterial cell membrane in order to act as a channel for permeation of cations across the membrane; this disturbed the ionic balance and ultimately led to the observed Gr antibiotic activity (Harold and Baarda, 1967; Clement and Gould, 1981; Hamada et al., 2010). Thus, for the DODAB/Gr combinations, the mechanism involved would include both the lytic aspects of DODAB interaction with the bacteria and the Gr effects on membrane function and selectivity in the transport of ions and nutrients and ion distribution in the cell.

DODAB could be incorporated in a polymeric biocompatible network of PMMA (Pereira et al., 2008) but displayed limited antimicrobial activity therein (Table 1) in contrast to the one of the more mobile CTAB surfactant which readily diffused across the polymeric PMMA network, reached attached or free bacteria and displayed good antimicrobial activity (Melo et al., 2011). Therefore, the good miscibility of DODAB lipid in the polymeric network of PDDA hampered DODAB diffusion to the outer medium where DODAB would act against the bacteria.

*L. monocytogenes* was very sensitive to the cationic lipid DODAB and the antimicrobial neutral peptide Gr (Table 1). Lysozyme and cationic peptides targeting the *L. monocytogenes* cell wall to promoted bacterial lysis. The introduction of specific modifications in components of the cell envelope as a strategy developed by bacteria rendered them undetectable to both immune recognition and to the bacteriolytic activity of host defense enzymes such as lysozyme and cationic antimicrobial peptides (Davis and Weiser, 2011; Carvalho et al., 2014). It seems that *L. monocytogenes* did not develop yet any mechanism against DODAB or Gr so that these might be advantageously employed in anti- *L. monocytogenes* coatings. On the other hand, the cationic antimicrobial polymer PDDA, similarly to cationic peptides did not affect this pathogen (Table 1). This is understandable from the already disclosed *L. monocytogenes* mechanisms to fight the cationic antimicrobial peptides. Curiously, the spherical assembly of PDDA as an outer shell of a PMMA/PDDA NP exhibits a reduction of 5 logs against *L. monocytogenes* (Table 1), suggesting that this bacterium is not prepared against this cationic NP and this also may become an asset in the fight against the pathogen.

Alternating layers of branched polyethylenimine and styrene maleic anhydride copolymer were applied onto the surface of polypropylene yielding coatings with low surface energy and enhanced antimicrobial character due to the presence of both cationic and N-halamine moieties; the coating inactivated *L. monocytogenes* by ~3 logarithmic cycles whereas in the form of N-halamines there was more than 5 logarithmic cycles in the viable cells counting (Bastarrachea and Goddard, 2015). In this respect, it seemed advantageous to introduce PMMA/PDDA NPs as efficient assemblies to reduce *L. monocytogenes* cell viability by 5 logarithmic cycles (Table 1).

*S. enterica* is one of the most important foodborne pathogens, leading to millions of cases of enteric diseases, thousands of hospitalizations and deaths worldwide each year (Hur et al., 2011). These bacteria were not sensitive to the majority of the cationic assemblies tested (Table 1) with exception of PDDA (3 logs reduction in viability) or PMMA/PDDA (5 logs reduction in viability) (Table 1). Although the antibacterial effect of antimicrobial peptides and polymers was mediated by membrane disruption with leakage of intracellular compounds (Carrasco et al., 2015), it was not clear how they reached the bacterial cytoplasmic membrane, crossing barriers such as the external membrane of Gram-negative bacteria and the cell wall of Gram-positive bacteria. Possibly the peptide or polymer first targets the outer cell wall and then undergoes a self-promoted uptake (Hancock, 1997; Yaron et al., 2003). In this respect, our results suggested that only PDDA and PMMA/PDDA NPs targeted the cytoplasmic membrane of *S. enterica* causing lysis and death. In particular, the activity of the NPs was higher than the one of the free polymer (Table 1), suggesting that they were more effective in inducing membrane disruption than the free polymer.

## Author contributions

LC and RB performed all the experiments, analyzed the results and helped writing the manuscript; RR provided technical assistance and helped discussing the manuscript; JS provided all bacterial strains and important advice for growing them; AC designed the study, interpreted the results and wrote the manuscript.

## Funding

Financial support was from Conselho Nacional de Desenvolvimento Científico e Tecnológico (CNPq 302352/2014-7).

### Conflict of interest statement

The authors declare that the research was conducted in the absence of any commercial or financial relationships that could be construed as a potential conflict of interest.

## Abbreviations

ATCC: American Type Culture Collection
BF: Bilayer fragments
CFU: Colony forming unit
CTAB: cetyltrimethyl ammonium bromide
DLS: Dynamic light scattering
DODAB: Dioctadecyldimethylammonium bromide
Dz: zeta-average diameter
Gr: Gramicidin D
LV: Large vesicles
MBC: minimal bactericidal concentration
MIC: Minimal inhibitory concentration
MHA: Mueller-Hinton agar
NP: nanoparticle
P: Polydispersity
PDDA: poly (diallyldimethylammonium) chloride
PMMA: poly (methylmethacrylate)
TFE: 2,2,2-trifluoroethanol (TFE)
ζ: zeta-potencial.
